# Increased frequency of TIGIT+ CD4 T Cell subset in autoantibody-positive first-degree relatives of patients with rheumatoid arthritis

**DOI:** 10.3389/fimmu.2022.932627

**Published:** 2022-07-28

**Authors:** Vidyanand Anaparti, Stacy Tanner, Christine Zhang, Liam O’Neil, Irene Smolik, Xiaobo Meng, Aaron J. Marshall, Hani El-Gabalawy

**Affiliations:** ^1^ Manitoba Center of Proteomics and Systems Biology, Departments of Internal Medicine, University of Manitoba, Winnipeg, MB, Canada; ^2^ Department of Immunology, University of Manitoba, Winnipeg, MB, Canada; ^3^ Rheumatic Diseases Unit, Department of Internal Medicine, University of Manitoba, Winnipeg, MB, Canada

**Keywords:** TIGIT, PD-1, rheumatoid arthritis, first-degree relatives (FDRs), multicolor flow cytometry (MFC), immunophenotyping analysis

## Abstract

**Background:**

Despite immune cell dysregulation being an important event preceding the onset of rheumatoid arthritis (RA), the phenotype of T and B cells in preclinical RA is less understood. The aim of this study was to characterize T and B cell populations in RA patients and their autoantibody (aAb) negative and positive first-degree relatives (FDR).

**Methods:**

Cryopreserved peripheral blood mononuclear cells (PBMCs) collected at scheduled visits from aAb-(n=25), and aAb+ FDR (n=10) and RA patients (n=13) were thawed and stained using optimized antibody cocktails as per a specific 13-color T or B cell panel. Immunophenotyping was performed using a Cytoflex LX (Beckman-Coulter) flow cytometer and FlowJo software was used for analyzing the frequency of immune cell populations.

**Results:**

Multicolor flow cytometry experiments identified an increased TIGIT expression in circulating lymphocytes of aAb+ FDR and RA patients, relative to aAb- FDR (P<0.01). These TIGIT^+^ T cells exhibited a memory phenotype and expressed high levels of PD-1, ICOS, HLA-DR, CXCR3 and CXCR5. Moreover, increased TIGIT^+^ CD4 T cell frequency correlated with the frequency of PD-1^+^ CD4 T cells (r = 0.4705: *P* = 0.0043) and circulating levels of ACPA and RF. We also identified a decreased frequency of CD27+IgD- switched memory B cells in RA patients (*P* < 0.01), while increased frequency of TIGIT+ CD4 T cells in FDR correlated with the frequency of PD1^+^PTEN^+^ B cells (r = 0.6838, *P* = 0.0004) and autoantibody positivity (*P* = 0.01).

**Conclusion:**

We demonstrate TIGIT as a distinct CD4 T cell marker for differentiating aAb- FDR from aAb+FDR and might play a critical role in regulating T and B cell crosstalk in preclinical RA.

## Introduction

Rheumatoid arthritis (RA) is a systemic autoimmune disease characterized by chronic, immune mediated synovial inflammation leading to cartilage and bone destruction, joint deformity, and functional loss (1). Considerable evidence has been accumulated to suggest that seropositive RA evolves as a continuum involving distinct preclinical phases of systemic autoimmunity and immune dysregulation occurring prior to onset of clinically classifiable inflammatory arthritis (IA) (1). Currently, this preclinical phase of RA is primarily characterized by the detection of circulating RA-associated autoantibodies such as anti-citrullinated protein antibodies (ACPA) and rheumatoid factor (RF), in some cases these being detected years before the onset of clinically detectable joint inflammation (1, 2).

The immunological basis for the development of RA autoantibodies during the preclinical phase, and the maturation of the autoimmunity to ultimately become pathogenic, remains an area of considerable interest and research activity. Based on our current understanding, these immunological processes require complex interactions between T and B lymphocyte populations that involve specific cognate recognition of autoantigens, as well as antigen-independent regulatory mechanisms (3). As such, it has been proposed that an expanded pathogenic CD4^+^ T-cell population mediates activation of autoreactive B cell recognizing post-translationally modified self-antigens and resulting in the production of matured autoantibodies directed towards these autoantigens (4, 5). These processes typically occur in germinal centers of lymphoid tissues and involve a well characterized population of T follicular helper cells (Tfh) expressing CXCR5 and PD-1 (6). Of considerable interest has been the demonstration of a distinct population of activated CD4+ T cells, both in the blood and the affected synovial tissues, characterized by PD-1^hi^CXCR5^-^ expression, and labelled as peripheral-helper T cells (Tph cells) (7). This unique population demonstrates an increased expression of IL-21 (interleukin 21), CXCL13, and ICOS, these being involved in B-cell differentiation, migration to the inflamed tissues, and autoantibody production within the synovium (7). Tph cells also express TIGIT, an immune checkpoint receptor expressed primarily on regulatory T cells, activated T cells, B cells and natural killer cells and inhibits effector T cell responses (8). Importantly, it has been demonstrated that there is an increased frequency of these PD-1^hi^CXCR5^-^ Tph cells in patients with early-RA, active-RA, and even those in clinical remission (7, 9). Moreover, recent studies showed that during the preclinical phase there was a decreased frequency of naïve and regulatory T cells, along with an increased frequency of inflammation-related cells (10, 11). Indeed, it has been proposed that the CD4+ T cell dysregulation could be utilized as a predictor of progression towards disease development (10, 11).

Our aim of this study was to identify the differences in the phenotype of T and B cell populations in autoantibody-positive (aAb+) first-degree relatives (FDR) of RA patients, compared to both unaffected FDR without detectable RA autoantibodies (aAb- FDR), as well as to RA patients with established disease. We hypothesized that individuals with detectable RA autoantibodies, but no clinically evident disease represent an intermediate phenotype exhibiting some of the immunological features seen in the circulating lymphocytes of patients with established RA.

## Methods

### Study design

To better understand the preclinical stages of RA, we have assembled a cohort of FDR of Indigenous North American (First Nations, FN) RA patients, this population being known to have a high prevalence of RA autoantibodies, and to have an increased risk of future RA development (12, 13). Study participants were recruited from urban and rural First Nations (FN) communities in Central Canada. The Research Ethics Board of the University of Manitoba approved the overall design of the study and consent forms (HS2005:093, HS14453; Early Identification of Rheumatoid Arthritis in First Nations). The conduct of the study was guided by the Tri-Council Policy Statement: Ethical Conduct for Research Involving Humans – TCPS 2 (2018) Chapter 9: Research Involving the First Nations, Inuit and Métis Peoples of Canada and the principles of Community Based Participatory Research, a cornerstone of the Canadian Institutes of Health Research guidelines for Aboriginal health research (http://www.cihr-irsc.gc.ca/e/29134.html). This First Nations prospective longitudinal study includes oversight from Indigenous community members and Elders and includes signed research agreements, developed through mutual collaborations with First Nations communities. Additionally, an Arthritis Advisory Committee, with Indigenous community representatives collaborates on proposed research work. Study participants provided free, informed consent. Within this cohort of FDR, we identified individuals in whom ACPA and/or RF were detectable, and in a subset of these individuals for whom peripheral blood mononuclear cells were available for study.

This sub-study was undertaken at a single timepoint in three distinct groups: (1) aAb+ RA patients (ACPA and/or RF), all of whom met the 2010 ACR/EULAR criteria (n=13), (2) aAb+ FDR with detectable ACPA or RF titers (>20 U/mL), but without any clinical evidence of joint or systemic inflammation (n=10), and (3) unaffected aAb- FDR (n=25) without any arthralgia, systemic inflammation and detectable ACPA or RF titers. RA patients and FDR studied here were, in most cases, not related. As such, these groups represent a hypothesized continuum, where the aAb+ unaffected group represents an intermediate phenotype that, in some cases, leads to the future development of clinically classifiable RA. Characteristics of the study participants are shown in **Table 1**.

**Table 1:** Baseline characteristics of the study population: All values are reported as either mean (SD) or n (%).

	FDR		RA(n=13)
	aAb-(n=25)	aAb+(n=10)	
**Age, years, mean (SD)**	43.6 (10.1)	47.7 (11.2)	44.1 (12.5)
**Female, n (%)**	11 (44)	4 (40)	9 (69.23)
**CRP, mg/L, mean (SD)**	2.7 (1.9)	3.7 (3.7)	15.5 (13.4)^$^
**BMI, kg/m2, mean (SD)**	28.9 (6.5)	29.3 (5.5)	28.3 (5.4)
**DAS28, mean (SD)**	–	–	3.9 (1.5)
**Ever Smokers (%)**	22 (88)	9 (90)	10 (77)
Autoantibody(aAb) status*			
RF+ only (%)	0	3 (30)	1(7.7)
ACPA+ only (%)	0	3 (30)	1 (7.7)
ACPA+ and RF+ (%)	0	4 (40)	7 (53.8)
ACPA-/RF- (%)	25 (100)	0	2 (15.4)

*Autoantibody status is available for two RA patients.

^$^ = *P*<0.05 compared to aAb- or aAb+ FDR; analyzed by Mann-Whitney test. RA, Rheumatoid Arthritis; FDR, first-degree relative; aAb, autoantibody; CRP, C-reactive protein; RF, rheumatoid factor; ACPA, anti-citrullinated protein antibody; BMI, body mass index; DAS28, disease activity score 28.

### Sample collection, storage, serology and PBMC isolation

Venous blood was collected into BD Vacutainer SST™ serum separation tubes (BD Biosciences, US) by a trained phlebotomist or study nurse, allowed to clot for 35 minutes and then centrifuged to separate the serum. Aliquots of sera were stored at −20°C until required for serology assays. C-reactive protein (CRP) levels were measured using a human high-sensitivity CRP (hs-CRP) ELISA kit (Biomatik, Canada) as per the manufacturer’s instructions. ACPA titer was determined using either the anti-CCP2 on a BioPlex^®^ 2200 System (Bio-Rad, US) or anti-CCP3 kits (Inova Diagnostics Inc, San Diego, CA). ACPA seropositivity status was considered negative if the titer was below manufacturer’s standardized assay cutoff (< 20U/mL, also known as upper limit normal or ULN) (13). For peripheral blood mononuclear cells (PBMC), venous blood was collected into heparinized vacutainers (BD) and isolation was performed using SepMate™ tubes (StemCell Technologies) as per the manufacturer’s protocol. Isolated PBMCs were cryopreserved in a freezing medium (90% fetal bovine serum and 10% DMSO) and stored in liquid nitrogen.

### Multi-color flow cytometry

T and B cell immunophenotyping was performed by multicolor flow cytometry on frozen PBMCs. Cells were thawed, washed with PBS + 1%BSA solution and surface-stained for either T or B cell markers at 4°C for 30 minutes. Stained cells were run on Cytoflex LX (Beckman-Coulter). OneComp eBeads™ (Thermofisher Scientific) were used as compensation controls, while FMO (fluorescence minus one) controls were used as negative controls. CD4^+^ T cells were represented as percent of CD3^+^dump^-^ (CD19^+^/CD14^+^/CD56^+^/viability) population, while B cells were represented as a percent of CD19^+^dump^-^ (CD14^+^/CD56^+^/CD3^+^/viability) population. Antibody details are listed in the **Supplementary Table S1**. CD4^+^ T cell and CD19^+^ B cell subset gating strategy is presented in **Supplementary Figure S1**.

### Data analysis & statistics

Flow cytometry data was analyzed by FlowJo (v10.8). Graphs and statistics were generated using GraphPad Prism (v9.1). Compensated fcs (flow cytometry standard) data files were pre-processed using CytoNorm plugin for batch normalization and FlowClean plugin to remove unwanted events (14, 15). Gates were set to define a population subset using FMOs as negative control. Mann-Whitney U test, Kruskal-Wallis test with Dunn’s *post-hoc* correction, Wilcoxon matched-pairs signed test, Spearman rank correlation analyses or regression analyses were used for statistical comparison as per the requirement. *P*-values < 0.05 were considered as statistically significant.

## Results

### Study population

**Table 1** outlines the characteristics of these three groups. There were no significant differences in age, sex, BMI, or smoking frequency between the groups. All the RA patients met 2010 ACR criteria, had established disease with a mean DAS28 score of 3.9 ± 1.5, and were receiving stable doses of disease-modifying anti-rheumatic drugs. Of these RA patients, 53.8% (7/13) were seropositive for both ACPA and RF, 7.7% (1/13) were either positive for ACPA or RF and 15.4% (2/13) were negative for both ACPA and/or RF. Autoantibody data was unavailable for 2 RA patients. Neither of the two FDR groups had clinically detectable RA, although as we previously reported, joint symptoms were prevalent in this population irrespective of their seropositivity status (16). Of note, the aAb+ FDR group was comprised of 3 individuals who were positive for ACPA only (222.5 ± 83.2 U/mL; mean ± SD), 3 individuals who were positive for RF only (27.9 ± 8.9 IU/mL; mean ± SD), and 4 individuals who were positive for both ACPA (59.6 ± 21.9 U/mL) and RF (231.5 ± 331 IU/mL).

### RA patients and aAb+ FDR have increased frequency of TIGIT-expressing CD4^+^ T cells

**Figure 1:**
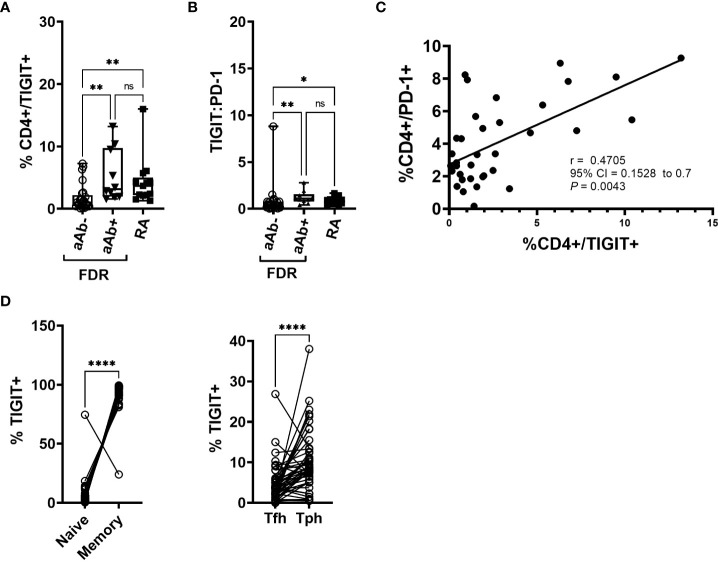
**(A)** Box-whiskers plot showing the frequency of CD4+ cells positive for TIGIT between aAb-FDR (n=25), aAb+FDR (n=10) and RA patients (n=13). *P<0.05, **P<0.01; Data was analyzed using Kruskal-Wallis method with Dunn’s *post-hoc* test. **(B)** Box-whiskers plot showing TIGIT: PD-1 ratio between aAb-FDR (n=25), aAb+FDR (n=10) and RA patients (n=13). *P<0.05, **P<0.01; Data was analyzed using Kruskal-Wallis method with Dunn’s *post-hoc* test. **(C)** Figure showing Spearman rank correlation plot between the frequency of TIGIT+ vs PD-1+ CD4 T cells. **(D)** Plot showing the frequency of naïve, memory, Tph and Tfh cells in the TIGIT+ fraction in all the subjects (n=48). Data analyzed by Wilcoxon matched - pairs signed rank test. ****P<0.0001. ns, non-significant.

Relative to aAb-FDR, RA patients and aAb+ FDR had a significantly higher frequency of TIGIT^+^ CD4 T cells and TIGIT: PD-1 ratio (*P* < 0.01; **Figures 1A**, **B**; **Figure S1** shows gating strategy of TIGIT^+^ CD4 T cells). Frequency of TIGIT^+^ and PD-1^+^ CD4 T cells was used to calculate the ratio. We also observed a strong correlation between TIGIT^+^ and PD-1^+^ CD4 T cells in FDR (*P* = 0.0043; **Figure 1C**). We observed that ~93% of memory (CD4^+^ CD45RA^-^) T cells were TIGIT^+^ (**Figure 1D**) and were found at a higher frequency in the Tph fraction (CXCR5^-^PD-1^hi^) than in the Tfh fraction (CXCR5^+^ PD-1^hi^; mean 10.18 vs 4.723; *P* < 0.001; **Figure 1D**). Expression of other phenotypic markers on the surface of peripheral blood CD4^+^ T cells was similar between aAb-FDR and aAb+FDR and RA patients (**Figure S2**). We also did not observe any significant differences in the proportion of major memory CD4^+^ T cell subsets between the two groups based on CCR7 or CD45RA expression (**Figure S2**).

**Figure 2:**
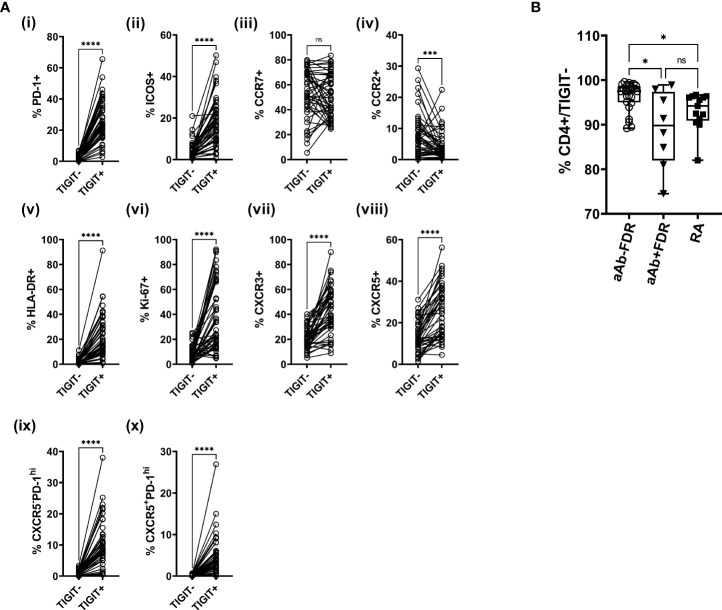
**(A)** Plots showing the frequency of TIGIT^+^ and TIGIT^-^ CD4 T cells expressing various phenotypic markers in the entire study population (i -x; n = 48). Data was analyzed using Wilcoxon matched pairs signed rank test. ***P<0.001, ****P<0.0001 ns, non-significant. **(B)** Box-whiskers plot showing the frequency of TIGIT^-^ CD4+ cells between aAb-FDR (n=25), aAb+FDR (n=10) and RA patients (n=13). *P<0.05; Data was analyzed using Kruskal-Wallis method with Dunn’s *post-hoc* test. ns, non-significant.

Characterization of TIGIT^+^ CD4 T cells across the entire study population showed that a higher proportion of these cells were positive for HLA-DR, Ki-67, PD-1, ICOS, CXCR3 and CXCR5, compared to TIGIT- CD4 T cells (**Figure 2**). CCR2 shows the opposite trend, with TIGIT^+^ cells showing significantly lower expression of this regulatory T cell-associated marker (**Figure 2A**). Interestingly, the TIGIT^+^ population shows selective phenotypic differences between aAb+ and aAb- FDR groups, with lower frequencies of Ki-67 and HLA-DR expression (**Figure S3**). While we observed a decrease in the frequency of TIGIT- CD4 T cells in aAb+FDR and RA patients relative to aAb-FDR (**Figure 2B**), these cell subsets did show any difference in the expression of phenotypic markers such as Ki-67 and HLA-DR (**Figure S4**). Taken together, these findings suggest that TIGIT^+^ generally cells have a higher activation status, exhibit a stronger proliferation index and better migratory capacity compared to TIGIT- cells. Expression of some activation and proliferation markers on TIGIT^+^ CD4 T cells is lowered in autoantibody-positive FDR, potentially indicating that chronic stimulation leads to a decreased activation status and a reduced proliferative capacity.

### Frequency of TIGIT^+^ CD4 T cells in FDR correlate with the frequency of PD-1^+^ and PTEN^+^ B cells

**Figure 3:**
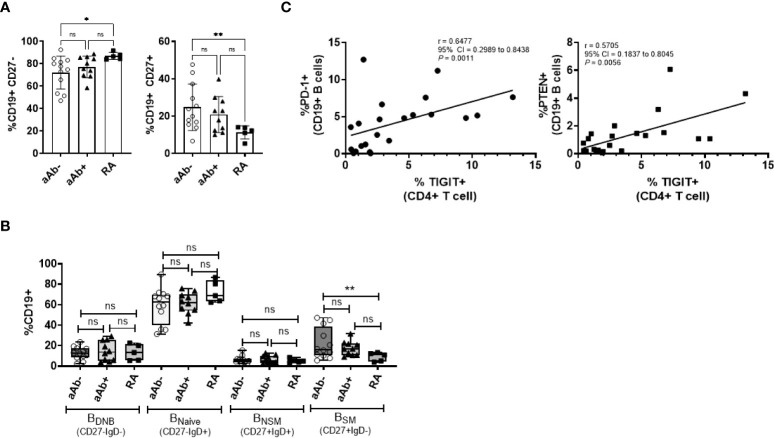
**(A)** Box-whiskers plot showing the frequency of CD19^+^CD27^-^ (naïve) and CD19^+^CD27^+^ (memory) B cells between aAb-FDR (n=12), aAb+FDR (n=10) and RA patients (n=5). **(B)** Box-whiskers plot showing the frequency of B-cell subsets between aAb-FDR, aAb+FDR and RA patients. **(C)** Spearman correlation plots showing the relationship between the frequency of TIGIT+ CD T cells (x-axis) and frequency of PD-1^+^ or PTEN^+^ (y-axis) in FDR (n=34). Data was analyzed by Kruskal-Wallis test with Dunn’s *post-hoc* analysis. *P<0.05, **P<0.01, ns, non-significant.

TIGIT expression on CD4 T cells facilitates T-B cell interactions and promotes B-cell differentiation into antibody-secreting plasmablasts (8). Therefore, we next analyzed the phenotype of CD19^+^ B cells in the peripheral blood of a subset of FDR (both aAb- and aAb+) and RA patients on whom CD4 T cell phenotyping was done (**Figure 3** and **S5**). Relative to aAb-FDR, RA patients demonstrated a high frequency of CD27^-^ naïve B cells. The frequency of CD27^+^ memory B cell population, including switched memory B cell subsets (B_SM_; CD27^+^IgD^-^) was lower in RA patients compared to aAb- FDR (**Figures 3A**, **B**). Further analysis of individual markers showed no significant differences between the three groups (**Figure S5**). However, we observed a strong correlation between the frequency of TIGIT^+^ CD4 T cells and PD-1^+^ or PTEN^+^ B cells (*P* = 0.0011 and *P* = 0.0056 respectively, **Figure 3C**). The frequency of TIGIT^+^ CD4 T cells also correlated with PD1^+^PTEN^+^ B cells (r = 0.7322, 95% CI = 0.4045 to 0.8933, *P*=0.004) indicating an association between the expression of co-inhibitory receptors on T and B cells supposedly mediated through PI-3K signaling. Of note, no such correlation was observed in the RA patients.

## Discussion

Currently, the most widely accepted biomarker for determining the risk of future RA development is the detection of circulating RA autoantibodies, particularly ACPA and RF, in otherwise clinically unaffected individuals. We and others have shown that a proportion of these RA autoantibody positive individuals will ultimately develop clinically definable RA, albeit after a variable period of follow-up (2, 13). The immunological mechanisms that underlie the development of the RA autoantibodies, and the progression towards pathogenic autoimmunity in specific individuals, remain unclear. Although alterations in circulating cytokines and chemokines detected in readily accessible serum/plasma samples have provided important clues in this regard (3, 10, 17, 18), disturbances in immune cell populations have not been defined, primarily because of the difficulty in obtaining suitable samples from at-risk individuals. In the current study, we used multiparametric flow cytometry to profile peripheral blood T and B lymphocytes in a cohort of RA autoantibody positive at-risk individuals and compared them to autoantibody negative individuals from the same population, and to RA patients with established disease. Our results support the hypothesis that individuals with detectable RA autoantibodies, but no clinically evident disease represent an intermediate phenotype exhibiting some of the immunological features seen in the circulating CD4^+^ T lymphocytes of patients with established RA, particularly an increased expression of the immune checkpoint receptor TIGIT, primarily among PD-1^+^ cell subsets.

Our findings are consistent with those made by Ponchel et al. and others, suggesting that T and B cell dysregulation is observed in the preclinical stages of RA and supports the idea of using multiparametric T cell quantification as a clinical tool for predicting stages of preclinical RA (10, 11). ACPA are RF are insufficient in identifying individuals at a high-risk of disease progression as we know that there is a high likelihood of seroconversion to an autoantibody-negative state in those FDR who are non-progressors (13). As a result, there is an increased need of reliable biomarkers that can predict individuals at different stages along the RA continuum and help us identify individuals ‘at-risk’ of disease progression. Multi-parametric immune cell profiling allows us to stratify individuals along the RA continuum and allows us to develop hypotheses and explore mechanisms underlying these different stages of disease development, which can eventually lead to a targeted interventions (10).

Elevated TIGIT expression in aAb+ RA patients concurs with the observations made by Luo et al. and others, who found an increased TIGIT expression both in the peripheral and synovial CD4 T lymphocytes of RA patients (19–21). In RA patients, increased TIGIT expression correlated with disease activity, was an independent predictor of RA treatment response, and inhibited CD4 T cell effector responses such as cell proliferation and secretion of proinflammatory cytokines IFN-γ and IL-17 (19, 20). For the first time, we demonstrate an expanded TIGIT^+^ CD4 T cell subset in aAb+FDR, some of whom might progress towards developing inflammatory arthritis. Our study also indicates the presence of a crosstalk between the presence of circulating RA autoantibodies and TIGIT expression on CD4 T cells. In FDR, increased TIGIT expression on CD4 T cells, along with reduced CCR2, CXCR3 and Ki-67 expression on TIGIT^+^ CD4 T cells and correlation with ACPA and RF indicates an autoantibody-mediated polarization of CD4 T cells to either an exhaustive or a regulatory phenotype (11, 22). This hypothesis is supported by the observations made by Hunt et al. who demonstrated an expansion of regulatory T cells in at-risk seropositive individuals prior to onset of inflammatory arthritis (11). Most importantly, expression of TIGIT defines a distinct regulatory T cell population that exhibit an activated phenotype and suppress Th1 and Th17 cell differentiation and effector responses (23). Analysis of T cells in individuals who are prospectively followed till they develop inflammatory arthritis will provide us further insights into the functional role of TIGIT^+^ T cells in RA.

We demonstrated a strong correlation between TIGIT^+^ CD4 T cells and PD1^+^PTEN^+^ B cells (r = 0.7322, 95% PI = 0.4045 to 0.8933, *P* < 0.0004). PTEN and PD-1 are essential for maintaining peripheral B cell tolerance against autoantigens and mediate B cell functions such as antibody class switching, somatic hypermutation, migration into germinal centers in the synovium and memory B cell differentiation on plasmablasts (7, 21). In our study, we also observed an increased in TIGIT: PD-1 ratio in both RA patients and aAb+FDR along with a positive correlation between TIGIT positivity of T cells and the frequency of PD1^+^PTEN^+^ B cells. These findings point towards a novel regulatory function of TIGIT^+^ T cells in determining the fate of memory B cells in an environment that contains increased amounts of cytokines such as IL-2, IL-15, IL-12 and IFNα (8, 24). While functional studies are needed to demonstrate these events, we are also interested in exploring the antibody repertoire of increased CD27^+^ memory B cells (switched and non-switched), the relative percentage of those being a citrulline-reactive and the role of TIGIT in this process.

The strength of our study is the characterization of T and B cell immunophenotype in the same sample, which allows us to correlate and study the relationship between different immune compartments within the individual with acceptable confidence. A major limitation of our study is the lack of data that can demonstrate the function of TIGIT on T cells in the absence or presence of autoantibodies COVID-related logistical complications made it unfeasible for us to obtain PBMCs required for performing functional assays to assess the function of above-mentioned cell populations. These issues were also central to the low sample size observed in our study. We also acknowledge the fact that our study was performed on samples only from First Nations communities, which has been the primary focus of our research program owing to their increased genetic susceptibility to RA (13). Interestingly, irregular immune cell phenotype and an altered serum cytokine pattern observed in seropositive FDR concurs with the findings observed in other populations, thus advocating the need of undertaking many such studies on a larger sample size to better define immune cell perturbations during the preclinical period (5, 10, 11). Although long-term outcomes for these individuals would be of considerable interest, there was insufficient follow-up at the time of this manuscript to provide meaningful data.

In conclusion, we highlighted the role of co-inhibitory receptors on T and B cells, and their crosstalk with inflammatory cytokines in modulating adaptive immune responses to autoantigens during the preclinical stages of RA. Our findings provide compelling evidence showing a distinct preclinical immune activation in seropositive FDR, which is dependent on the appearance of RA-associated autoantibodies. Our study also found value in using T and B cell immune profiling as a clinical predictor of RA onset. Our future plan is to develop a model by combining immunophenotyping data, with serum cytokines and other known parameters of preclinical RA and evaluate its clinical applicability in a larger cohort of ‘at-risk’ people for RA prediction. We foresee a long-term use of such models as an outcome measure in intervention studies aimed at RA prevention.

## Data availability statement

The raw data supporting the conclusions of this article will be made available by the authors, without undue reservation.

## Ethics statement

The studies involving human participants were reviewed and approved by Research Ethics Board, University of Manitoba. The patients/participants provided their written informed consent to participate in this study.

## Author contributions

VA, ST, and HE-G conceived research concept; VA, ST and CZ performed experimental work, VA analyzed data, VA and LO’N prepared figures, CZ, LO’N, AM, and HE-G assisted in data analysis, IS assisted in patient recruitment, IS and XM assisted in sample acquisition and storage, and VA and HE-G. drafted, and revised the manuscript, and all the co-authors participated in editing the manuscript.

## Funding

The study was supported by individual grants to Dr. Hani El-Gabalawy from the Canadian Institutes of Health Research (CIHR MOP 77700). VA received Postdoctoral Fellowships from Research Manitoba and Arthritis Society of Canada.

## Acknowledgments

We acknowledge the contribution of study participants from rural and urban First Nations people who participated in this study. We also appreciate the support of Chiefs and Band Councils of Norway House Cree Nation and St. Theresa Point First Nation.

## Conflict of interest

The authors declare that the research was conducted in the absence of any commercial or financial relationships that could be construed as a potential conflict of interest.

## Publisher’s note

All claims expressed in this article are solely those of the authors and do not necessarily represent those of their affiliated organizations, or those of the publisher, the editors and the reviewers. Any product that may be evaluated in this article, or claim that may be made by its manufacturer, is not guaranteed or endorsed by the publisher.
