# Supplementary material for: Increased frequency of TIGIT+ CD4 T Cell subset in autoantibody-positive first-degree relatives of patients with rheumatoid arthritis
Source: Front Immunol. 2022 Jul 28;13:932627. doi: 10.3389/fimmu.2022.932627 (PMC9366176; doi:10.3389/fimmu.2022.932627)
Supplement: Supplementary file 1 [file DataSheet_1.docx]

**Supplementary Data Contents**

Figure S1: Gating strategy of TIGIT+ CD4 T cells

Figure S2: Frequency of T cell subsets between FDR and RA patients

Figure S3: Phenotype of TIGIT+ CD4 T cells between FDR and RA patients

Figure S4: Phenotype of TIGIT- CD4 T cells between FDR and RA patients

Figure S5: Phenotype of CD19+ B cells between FDR and RA patients

Table S1: List of antibodies used for multiparameter flow cytometry staining

Figure S1: (A) Gating strategy of TIGIT+ CD4 T cells


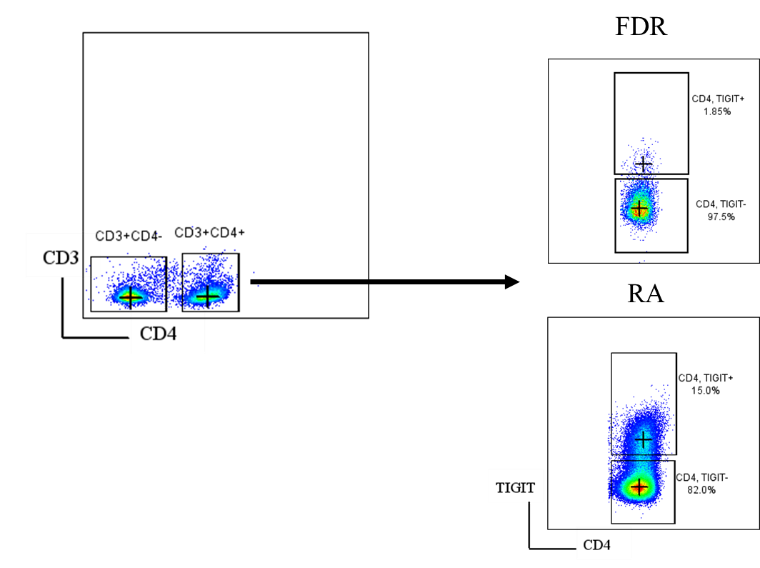


(B) Gating strategy of CD4 T cells based on CD45RA and CCR7


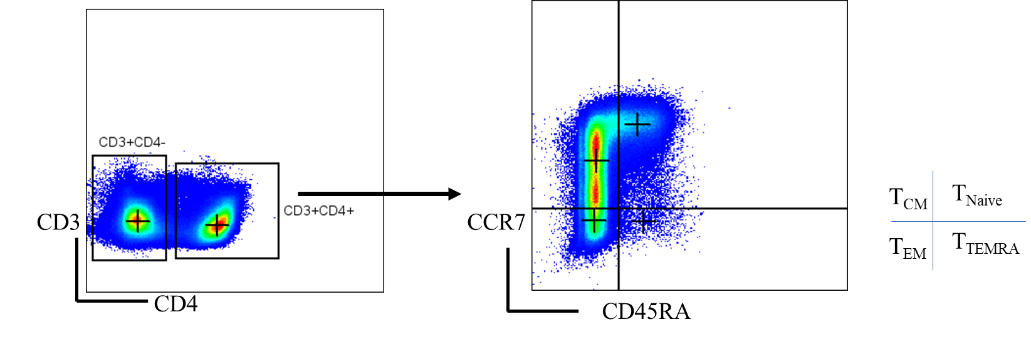


(C) Gating strategy of CD19+ B cells based on CD27 and IgD


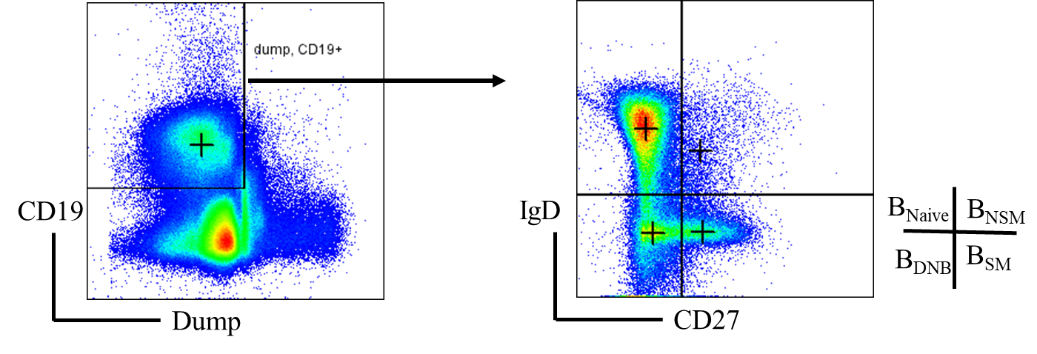


Figure S2: Box-whiskers plot showing the frequency of different T cell populations between aAb-FDR, aAb+FDR and RA patients. Data was analyzed using Kruskal-Wallis test with Dunn’s post-hoc analysis. **P*<0.05; ns=non-significant.

Figure S3: Box-whiskers plot showing the frequency of TIGIT+ CD4 T cells expressing different phenotypic markers between aAb-FDR, aAb+FDR and RA patients. Data was analyzed using Kruskal-Wallis test with Dunn’s post-hoc analysis. *P<0.05; **P<0.01; ****P<0.0001; ns = non-significant.

**Figure S4:** Box-whiskers plot showing the frequency of TIGIT- CD4 T cells expressing different phenotypic markers between aAb-FDR, aAb+FDR and RA patients. Data was analyzed using Kruskal-Wallis test with Dunn’s post-hoc analysis. *P<0.05; **P<0.01; ****P<0.0001; ns = non-significant

**Figure S5**: Box-whiskers plot showing the frequency of CD19+ B cells expressing different phenotypic markers between aAb-FDR, aAb+FDR and RA patients. Data was analyzed using Kruskal-Wallis test with Dunn’s post-hoc analysis. *P<0.05; **P<0.01; ****P<0.0001; ns = non-significant

Table S1: List of antibodies used for multiparameter flow cytometry staining

| T Cell Panel | | B Cell Panel | |
| --- | --- | --- | --- |
| Marker | Fluorochrome | Marker | Fluorochrome |
| CD19 (dump) | BV421 | CD3 | Pacific Blue |
| CD56 (dump) | BV421 | CD14 | Pacific Blue |
| CD14 (dump) | Pacific Blue | CD19 | APC-Cy7 |
| CD3 | APC-H7 | CD38 | BV711 |
| CD4 | AF700 | IgD | PerCp-Cy5.5 |
| CCR2 | FITC | CD27 | PE-Cy7 |
| CD45RA | V500 | CD80 | BB515 |
| CXCR3 | PE-CF594 | CD86 | BV510 |
| CXCR5 | PE | 9G4 | APC |
| PD-1 | BV605 | IgG | PE-CF594 |
| ICOS | BV650 | PD-1 | BV605 |
| HLA-DR | BV711 | PTEN | PE |
| Ki-67 | PerCp-Cy5.5 | Live/Dead | Pacific Blue |
| TIGIT | PECy7 |  |  |
| CCR7 | APC |  |  |
| Live/Dead stain | Pacific Blue |  |  |
